# Categorical emotion recognition from voice improves during childhood and adolescence

**DOI:** 10.1038/s41598-018-32868-3

**Published:** 2018-10-04

**Authors:** Marie-Hélène Grosbras, Paddy D. Ross, Pascal Belin

**Affiliations:** 1 Aix-Marseille Université, CNRS, Laboratoire de Neurosciences Cognitives, Marseille, France; 2 Department of Psychology, Durham University, Durham, United Kingdom; 3 La Timone Neuroscience Institute, Mixed Research Unit 7289 Centre National de la Recherche Scientifique and Aix-Marseille University, Marseille, France; 4 Département de Psychologie, Université de Montréal, Montréal, Québec, Canada

## Abstract

Converging evidence demonstrates that emotion processing from facial expressions continues to improve throughout childhood and part of adolescence. Here we investigated whether this is also the case for emotions conveyed by non-linguistic vocal expressions, another key aspect of social interactions. We tested 225 children and adolescents (age 5–17) and 30 adults in a forced-choice labeling task using vocal bursts expressing four basic emotions (anger, fear, happiness and sadness). Mixed-model logistic regressions revealed a small but highly significant change with age, mainly driven by changes in the ability to identify anger and fear. Adult-level of performance was reached between 14 and 15 years of age. Also, across ages, female participants obtained better scores than male participants, with no significant interaction between age and sex effects. These results expand the findings showing that affective prosody understanding improves during childhood; they document, for the first time, continued improvement in vocal affect recognition from early childhood to mid- adolescence, a pivotal period for social maturation.

## Introduction

Recognizing other people’s emotions is crucial for successful social interactions and for establishing intimate relationships throughout the lifespan^1,2^. This ability emerges early on and within the first half-year of life infants can respond to basic emotions from non-verbal cues. Yet infants’ reading of emotions is still rudimentary^3^ and it continues to refine throughout childhood^4,5^ and even adolescence e.g.^6–11^. Moreover, during late childhood and adolescence, the ability to identify emotions from non-verbal behavior (i.e. facial expression, body posture or tone of voice in speech) is related to academic achievement^12^, peer-rated popularity and quality of relationship with adults^13,14^, while being independent of IQ. Alteration of emotion processing in this age range has also been linked to a variety of developmental disorders such as autism^15^, attention deficit and hyperactivity disorder^16^, social anxiety^17^, psychopathy^18^, or conduct disorder^19^. It is thus of pivotal relevance to characterize the normal developmental trajectory of emotion perception during childhood and adolescence.

Most of the existing research on this topic, however, has focused on the perception of static facial expressions, presumably due to the assumption that faces are the most universal and reliable carriers of emotional signals. Some discrepancy exists between studies, but most show substantial improvement in facial expressions decoding skills until at least 10 years of age, with a number of studies indicating that, in more subtle detection tasks, 14 or 15 years old teenagers still do not perform as well as adults^4,7,9^. This line of research also points towards a small advantage of girls, across ages, in identifying nonverbal emotional cues rev. in^8,10,20^, with possibly an increase in the size of this advantage from childhood to early adulthood^21^.

In contrast, although the importance of voice as a medium of affective communication has been documented throughout history rev. in^22^, few studies have explored the development of emotion recognition in the auditory modality. This is surprising given the importance of voice in parent-child or peer-to-peer interactions, especially those taking place from a distance. Data indicate that infants can discriminate affect from tone of voice or from composite face-voice stimuli earlier than from faces alone^23–25^, leading researchers to suggest that in infancy voice is the primary channel for understanding others’ emotions. This is consistent with the earlier maturation of the auditory as compared to the visual system. Studies focusing on childhood, relying on different methodologies, have shown that children as young as four years old can label the emotions expressed by unfamiliar adults from their tone of voice and revealed substantial development between 4 and 11 years of age for the perception of basic emotions from the prosody in sentences^5,26–32^. Here the literature suggests that in this age range vocal expressions would be recognized equally well or with more difficulty than facial expressions^5,27,33–35^. Moreover, especially in pre-adolescents’ girls, abilities to process affective vocal expressions predict social competences better than facial expression recognition scores^14^. Given the extensive literature documenting a protracted development of face processing into adolescence, one can expect that full maturation of voice processing would thus also be occurring quite late. Yet, it is unclear whether emotional voice processing is adult-like at the end of childhood or whether maturation still occurs during adolescence: more research is needed with adolescents to provide a complete picture of development of emotion perception in the auditory modality (see meta-analysis in^21^). It is indeed increasingly recognized that important socio-emotional development occurs during adolescence^36^ in relation to changes in perceptual abilities^37^ and brain organization^38^. In particular, brain regions involved in voice and prosody processing, in the superior temporal and in the prefrontal cortex^39–41^, undergo structural and functional changes well into adolescence^38,42,43^.

Moreover, research so far has focused on how children process prosody in speech, which is confounded by the development of linguistic abilities. The processing of semantic content could have different interference effect at different ages, further confusing the interpretation of developmental findings^44,45^. Only a handful of studies have used auditory stimuli other than sentences to circumvent this issue, but have yielded partly discrepant results and only one has considered adolescents, but not beyond age 13^46^. Matsumoto and Kishimoto^47^ asked 50 4- to 9- years old children to select which amongst four emotions was expressed by a speaker naming individual letters or numbers with intonations depicting happiness, surprise, sadness or anger. They observed a substantial increase in correct recognition with age, with only minor differences across emotion categories. More recently, Allgood and Heaton^48^ used affect bursts, that is, short non-verbal vocal expressions such as laughter, weeping or scream^49,50^. They tested 228 children aged between 5 and 10 years and observed that 5- years old were significantly worse than 10-years old children when categorizing these stimuli as happy, sad or fearful. In neither of these studies, did the authors provide indication as to whether performance was different from adults. However, as in both studies 10 years old produced only 80% correct responses, one might expect that there is still improvement until adulthood. In contrast, Sauter *et al*.^29^ using the same material in addition to six others emotions (Anger, Disgust, Surprise, Contentment, Relief, Achievement) reported no significant age effect in a sample of 48 children of similar age (5–10 years), except for the identification of surprise, with again no comparison with adults’ data. It should be noted though that even their younger participants performed at surprisingly high level (overall 78% correct responses compared to 83% for the older children) leaving little room for improvement. In the same participants, however, recognition and categorization of emotion from inflected spoken words improved linearly for most emotions and was poorer than recognition from affect bursts in all age groups. Chronaki *et al*.^27^ used non-word interjections pronounced with three different emotions (happiness, anger and sadness) and manipulated with acoustic morphing to have different emotional intensities. They tested 98 children sorted into three groups (3–5; 6–9 and 10–11 years old respectively). They observed an improvement in the ability to discriminate these emotions between 3 and 11 years of age, with 11-years old exhibiting significantly poorer performance than an adult control group (73% accuracy vs 83% for the adults). In a subsequent study, they used meaningless sentences in different languages in a similar design and observed that early adolescents (age 11–13, n = 32) were not better than children (age 8–10, n = 25) at discriminating emotions, while they performed more poorly than adults (age 19–35, n = 22)^46^. Amongst these studies Allgood and Heaton^48^ and Chronaki and *et al*.^27^ observed a gender effect, consisting of better performance of females, albeit with apparently no interaction with age.

In summary, research on the development of vocal emotion perception using non-linguistic material is limited and provides fragmentary or inconsistent results regarding (i) the developmental trajectory, (ii) whether adults performance is reached by the end of childhood or whether this ability still changes during adolescence, a critical period for social adjustment, and (iii) whether there is any significant gender difference.

Regarding the latter question, it must be noted that gender effect in another domain, namely recognition of facial expressions, is small in children^20,51^, and might thus be undetected in individual smaller-scale studies. Moreover, in adults, females are faster and better at categorizing emotions from faces^10,52^ but also from voices^49,53^. The question of sex differences in the development of emotion perception in the auditory domain is thus worth more investigation.

Another factor that makes difficult comparing studies is the heterogeneity in testing procedures^51^. For example, Sauter and colleagues^29^ presented the audio stimuli on speakers and asked participants to select the corresponding emotion by choosing, on a printed sheet, a label illustrated with a photo depicting the facial expression. Responses were logged by the experimenter. Matsumoto *et al*.^47^ used the same procedure. Allgood and Heaton^48^ tested participants in groups in the classroom, playing the sounds on loudspeakers and asking children to circle, on a scoring sheet, the cartoon face corresponding to the emotion they though was displayed. Lastly, Chronaki *et al*.^27,46^ used a fully-computerized method, presenting the stimuli on headphones and asking participants to select amongst keyboard keys with emotional word labels printed on them (with the help of the experimenter for younger children). For the literature on facial emotions categorization, the oldest studies consist mostly of paper-and-pencil tests while more recent studies use mostly computerized task. Only one account, to our knowledge, shows that testing procedure did not impact developmental findings^7^. The question remains open with regards to affective vocalization processing.

The goal of the present study was to characterize the maturation of basic emotions identification from non-verbal vocalizations between childhood and adulthood. More specifically we aimed to confirm improvement in this ability during childhood and extend the investigation into adolescence. We asked 225 participants age 5–17 and 30 adults to recognize four discrete emotions (anger, happiness, sadness and fear) from short affective bursts in a forced-choice task. We expected to reproduce the findings of Mastumoto *et al*.^47^, Chronaki *et al*.^27^; Allgood and Heaton^48^ showing that recognition of emotion from non-linguistic vocalization improves throughout childhood. Further, we hypothesized that this improvement continues during adolescence. This would be in accordance with functionalist views of adolescent developments putting forward an adaptive refinement of social skills during this period of life^54^ and with the accruing data showing protracted maturation of the brain circuits involved in social and emotional signals perception rev. in^36^. In addition, in line with what has been described for faces^3,8^ and for prosody in children^27,29,34,45^, we expect different trajectories for the different emotions: happiness is likely to be recognized with adult’s accuracy at a younger age and sadness at a later age. Lastly, using a large enough sample we wanted to test whether the female advantage reported by Allgood and Heaton^48^ can be reproduced.

## Results

### Descriptive statistics

The average performance in the sample of children-adolescents was well above chance level (25%) with an average of 81.7% correct responses (median 85% correct, standard deviation 11.8%; skewness = −1.87, kurtosis = 9.73). The adults displayed an average score of 86.7 (median 87.5%, standard deviation 9.3; skewness = −0.42; kurtosis = 1.93). The difference between the two groups was statistically significant (Welsh t(253 = 2.67, CI of difference = [1.33 8.67], p = 0.011).

### Effect of testing procedure

In order to pool all participants into a single analysis, we aimed to verify first that there was no effect of testing procedure nor interaction with other factors. We first estimated a mixed-model in the children-adolescents sample, including the continuous factor age and categorical factors sex (male/female) and testing procedure (computer/ paper) with random intercept for subjects. There was no effect of testing procedure [Z-Wald = 0.2334; p = 0.81) nor interaction with the other factors (with age, Z = −0.179, p = 0.85; with sex: z = −0.19, p = 0.85). Removing this factor from the model did not alter the fit [Chi-square (4) = 0.75, p = 0.9447]. In addition, when we modeled separately data from the sample tested on computer and the sample tested on paper, with age and sex as factors, we observed similar parameter estimates in the two analyses and in the full model. Thus, the conclusions of our study hold whether we consider the full sample or only subsamples of participants tested with one or the other method. Therefore, for simplification we present now analysis of the full sample without the factor “testing procedure”.

### Effect of age and sex

The effect of age was highly significant (Z = 5.52, p = 3.4 × 10^−8^). Adding a quadratic term (age^2^) improved the model fit: Chi-sq(1) = 5.14, p < 0.023, indicating a non-linear developmental trajectory with faster improvement with age in childhood, followed by slower changes and plateau in late adolescence. The model with a cubic term did not converge. Based on the values predicted by the model at different ages (converted from logit to percent correct responses), the adults’ mean level of performance is reached between age 14 and 15.

**Figure 1:**
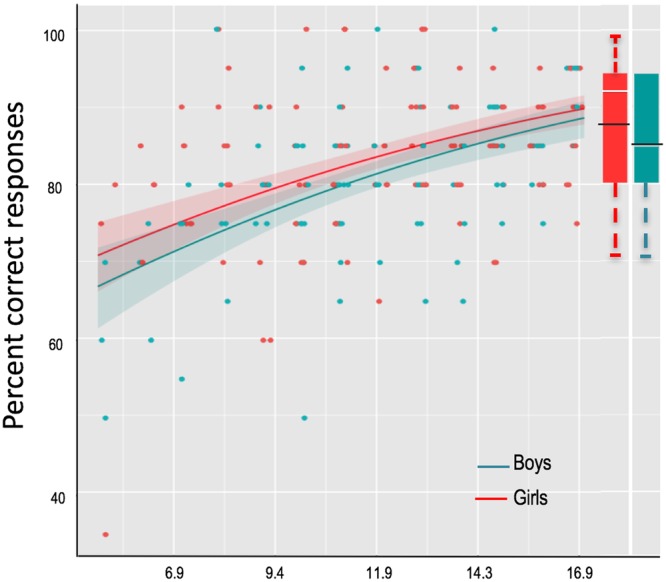
Proportion of correct responses as a function of age for boys and girls. Each dot represents the performance of one participant. The graph is centered on the mean age of the developmental sample (11.8 years) The lines represent the predicted performances as a function of age for males and females separately for the model: Response ~age + age^2^ + 1|Subject (from the function sjp.glmer; Lüdecke D (2017). *sjPlot: Data Visualization for Statistics in Social Science*. R package version 2.4.0, https://CRAN.R-project.org/package=sjPlot). Shaded areas represent confidence intervals. Boxplots represent median and 75 percentile in adults’ data; the black lines represent the mean performance of adults.

The effect of sex was also significant in the full model (Z = −2.017, p = 0.043). This was explained by females having significantly higher scores than males (83.2 +/− 6.2% *v*s 80.3 ± 6.7%, post-hoc t(223) = 3.27, CI = [1.1: 4.5]; p = 0.0012). There was no interaction between sex and age (comparison models with and without interaction term: Chi-square (1) = 0.021, p = 0.88), indicating similar age related trends in boys and girls. Looking at the model predictions separately for boys and girls showed that adults’ level of performance (mean for female adults: 88.6 +/− 9.9% correct; mean for male adults: 85 +/− 8.7%) is reached between 14 and 15 years of age in both sexes (Fig. 1).

### Effect of emotion

**Figure 2:**
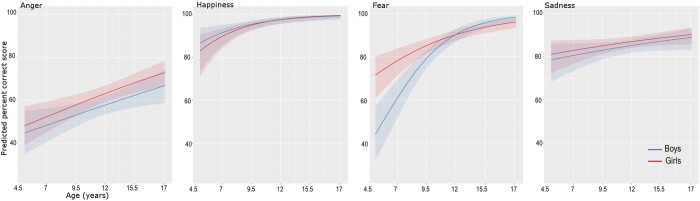
Logistic regression fitted for the four emotions separately. From left to right: Anger, Happiness, Fear and Sadness.

Including the factor Emotion in the mixed model logistic regression showed a highly significant effect of emotion (Z = 12.3, p < 10^−16^), in addition to the effects of age (Z = 6.6; p < 10–4), age-square (Z = −2.36, p = 0.018) and sex (Z = −2.025, p = 0.043). There was no interaction between emotion and sex (Z = 0.014, p = 0.98). The interaction between emotion and the linear effect of age was not significant (comparison models with and without interaction term: Chi-square (1) = 2.41, p = 0.12). The interaction between emotion and the quadratic term was significant (comparison models with and without interaction term: Chi-square (1) = 6.73, p = 0.00094). To explore this interaction, we fitted a logistic linear model on the percent correct recognition score for each emotion separately (Fig. 2 and Table 1). This revealed significant age-related effects for all the emotions with the quadratic term being significant only for sadness.

**Table 1:** Results from the mixed model logistic regression analyses for each emotion separately.

	Age	Age^2^	Sex	Age × Sex
ANGER	Chi = 16.23, P = 5.61 10^−5^	Chi = 0.03 P = 0.84	Chi = 3.21 P = 0.07	Chi = 0.04 P = 0.83
HAPPINESS	Chi = 4.88 P = 0.0271	Chi = 2.62 P = 0.10	Chi = 0.0.01 P = 0.91	Chi = 0.0027 P = 0.95
FEAR	Chi = 45.41 P = 0.16 10^−11^	Chi = 0.127 P = 0.72	Chi = 1.12 P = 0.28	Chi = 3.04 P = 0.08
SADNESS	Chi = 3.36 P = 0.066	P = 4.08 P = 0.043	Chi = 0.91 P = 0.34	Chi = 0.38 P = 0.56

The linear and quadratic effects of age, the effect of sex and interaction between age and sex effects are evaluated by comparing the fit of the logistic model with and without these factors^38^.

**Figure 3:**
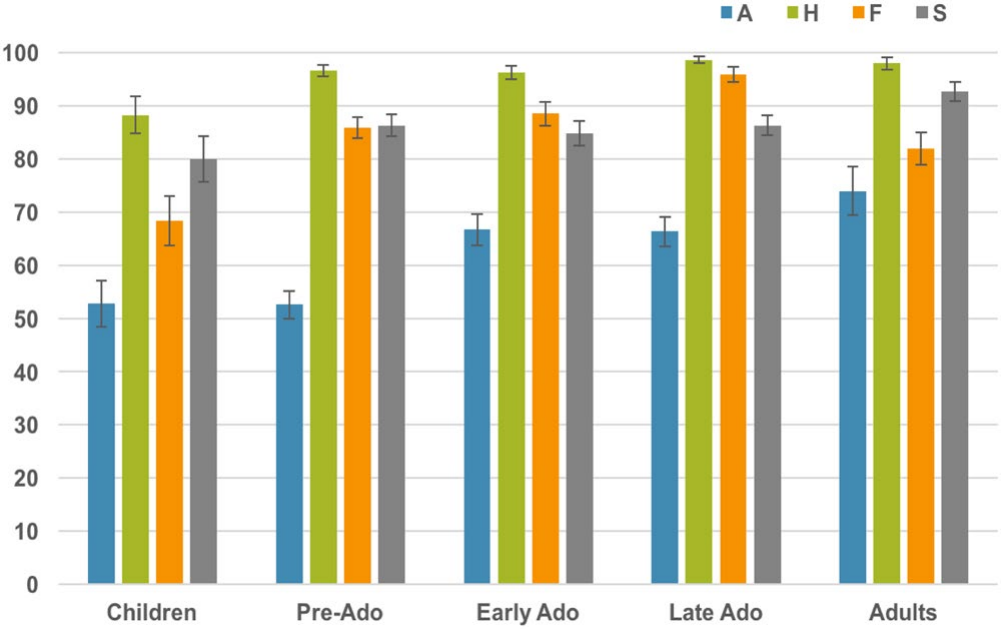
Proportion of correct responses (Mean and SEM) for each emotion separately for the five age groups (Children: 5–8 years old; early adolescents: 9–11 years old; mid-adolescents: 12–14 years old n = 54; late-adolescents: 15–17 years old and Adults).

Across the whole age range, happiness was the emotion recognized with the highest accuracy, followed by sadness and fear at around the same level while anger was the least well recognized (Fig. 3 and Table 3). Improvement within the age span was smaller for happiness and greatest for anger and fear. To characterize this effect, we compared the four emotions pairwise in five different age-groups (children, pre-adolescents, early adolescents, late adolescents and adults; see methods). The results are displayed in Table 2 and Fig. 3. Anger was significantly different from all the other emotions in all age-groups except in adults where it did not differ from fear. Happiness was significantly better recognized than all other emotions in pre- and early adolescents, but the difference between happiness and fear was not significant in adults and the difference between happiness and sadness was not statistically significant in adults and children. Performances for fear and sadness stimuli did not differ in the youngest age groups, but in late adolescents fear was better recognized than sadness and the reverse in adults.

**Table 2:** Comparisons of performance for pairs of emotions in four different age groups.

	Children (n = 41)	Pre-ado (n = 67)	Early ado (n = 54)	Late Ado (n = 63)	Adults (n = 30)
A < H	6.6 ***6.0*** × ***10***^***−4***^	13.1 ***1*** × ***10***^***−6***^	9;6 ***6.0*** × ***10***^***−4***^	12.3 ***6.0*** × ***10***^***−5***^	6.6 ***4.0*** × ***10***^***−4***^
A < F	2.9 ***0.013***	9.9 ***1*** × ***10−5***	5.48 ***4.0*** × ***10***^***−4***^	11.3 ***4.0*** × ***10***^***−5***^	2.1 *0.086*
A < S	5.1 ***5.75*** × ***10***^***−4***^	9.9 ***1*** × ***10***^***−5***^	5.95 ***5.0*** × ***10***^***−4***^	7.6 ***5.0*** × ***10***^***−4***^	4.8 ***8.0*** × ***10***^***−4***^
F < H	3.7 ***0.0011***	3.2 ***0.009***	4.57 ***9.0*** × ***10***^***−4***^	1.0 *0.311*	4.2 ***3.7*** × ***10***^***−4***^
F < S	1.3 0.061	0.09 0.99	−1.1 0.279	−3.7 ***4.0*** × ***10***^***−4***^	2.7 ***0.022***
S < H	1.5 0.124	4.1 ***0.012***	3.18 ***0.0023***	4.7 ***2.0*** × ***10***^***−4***^	1.4 0.17

Each cell indicates the t- value for the post-hoc comparison between pairs of emotion and, underneath, the corresponding p- value (Holm-Bonferoni correction), in BOLD if <0.05. A: Anger; H: Happiness; F: Fear; S: Sadness.

**Figure 4:**
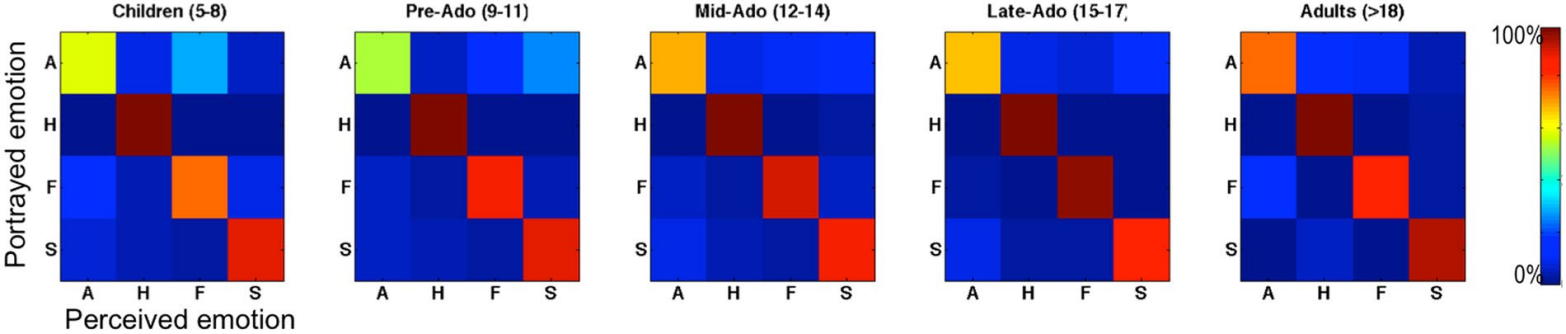
Confusion matrices for the five age groups. Each cell indicates number of times each emotion was chosen by participants (columns) as a function of the actual portrayed emotion (rows). A: Anger; H: Happiness; F: Fear; S: Sadness.

Second, we looked at how each emotion was miscategorized for each of the others, in the incorrect trials. Figure 4 and Table 3 show the confusion matrices in the four age groups and in adults. We observe relatively similar patterns of confusion across development. Anger was miscategorized most often as fear by children aged 5–8, and as sadness by adolescents. Fearful vocalizations were also sometimes miscategorized as angry by younger children as well as adults, while this confusion was less apparent in adolescents. Instead, adolescents showed a tendency to categorize sad vocalization as angry.

**Table 3:** Percentage of responses from the 4 emotions categories for each portrayed emotion. Diagonal represent the percent correct recognition.

Mean performance	Children (age 5–8)				Pre-Ado (age 9–11)				Mid-Ado (age 12–14)				Late-Ado (age15–17)				Adults			
	73.3				80.4				84.1				86.8				86.7			
Emotion	Perceived				Perceived				Perceived				Perceived				Perceived			
**Portrayed**	A	H	F	S	A	H	F	S	A	H	F	S	A	H	F	S	A	H	F	S
**Anger**	**52.7**	10.2	28.3	8.3	**52.5**	5.9	16.1	25.1	**66.7**	7.8	10	16.0	**66.3**	9	6.3	18.3	**74.0**	12.7	9.3	4.0
**Happiness**	3.9	**88.3**	4.4	3.4	1.2	**96.7**	0.9	0.9	0	**96.3**	1.5	1.8	0.33	**98.7**	1.0	0	0	**98**	0	2
**Fear**	14.1	6.3	**68.3**	10.7	6.3	3.6	**85.9**	4.5	4.8	1.8	**88.5**	4.8	2.7	0.33	**96.0**	1	14.0	1.3	**82.0**	2.7
**Sadness**	9.3	6.3	5.4	**80.0**	5.7	5.4	2.9	**86.2**	7.8	4.4	2.9	**84.8**	9	1.67	3	**86.3**	0.67	5.3	1.3	**92.7**

In the top line is the mean performance across the four emotions.

A: Anger; H: Happiness; F: Fear; S: Sadness.

## Discussion

We report for the first time the developmental trajectory of emotion perception from voice during childhood and adolescence. In a sample of 225 participants spanning an age-range from 5 to 17, we show that the ability to identify emotion from short affective bursts, in a forced-choice task, improves slowly but significantly with age, reaching adults’ level around the age of 15. The developmental curve is better characterized by a non-linear function, with faster improvement during childhood followed by slower changes and plateau during adolescence. Across ages, female participants recognized emotion slightly more accurately than male participants with no interaction between age and sex effects. Whether participants were tested on individual computers or in small groups with response given on paper did not affect performance nor the observed developmental trajectory. We discuss these findings and their implications in relation to the existing literature on the maturation of non-verbal signals perception.

First, our findings significantly expand the literature on affective bursts recognition during childhood. They comfort, with a different population and material, the results published by Matsumoto *et al*.^47^, Chronaki *et al*.^27^ and Allgood and Heaton^48^, who reported an improvement in vocal affect recognition between 5 and 10 years of age. In contrast, Sauter *et al*.^29^ did not observe any developmental differences in this age range, except for the emotion surprise, when participants had to categorize short vocalizations such as screams or laughers. In this latter study, which had a much smaller sample size, the young participants displayed very high accuracy, leaving little room for improvement. Maybe the stimuli employed appeared exaggerated and were therefore recognized better by younger children; the same participants had more difficulty identifying emotion from single-words (digits) inflected speech and, in this case, a difference between younger and older participants was apparent. This concurs to establish that maturation of affective voice processing takes place during childhood, although further investigation would be needed to characterize the acoustical parameters that facilitate emotion recognition by young children. It must be noted that the stimuli used in our study, taken from the Montreal Affective Voices database, have been validated for their ecological value, their reliability and for yielding similar intensity judgments as emotional stimuli from standard databases of facial and bodily expressions^55^.

In addition, here we show that the improvement in vocal affect recognition continues beyond childhood into adolescence, and we are able to model this trajectory. Such protracted development is unlikely to be linked to age-related changes in the processing of low-level acoustic cues that differs across emotions. Indeed, the ability to process frequency variations or pitch, which differentiate these emotions^49,56^, has been reported to be adult-like in children aged about 5^57^. Nonetheless, late development has been also observed in other aspects of voice processing: Mann *et al*.^58^ reported that the ability to recognize familiar voices and to form new memories of voices improved until 14 years of age. Some studies investigating sentence prosody recognition also suggest an improvement during adolescence: pooling together data from several studies using one of the most standardized measures of receptive nonverbal communication^34^ and its version using children voices as stimuli (DANVA2-CP), points towards a linear improvement in affective prosody identification between 4 and 19 years Table 2^59^. This is in agreement with a recent study showing that 13–15 years old were outperformed by adults for the recognition of affective sentence prosody, even more so in the more difficult condition of decoding emotion from sentences spoken by children^60^. It is also consistent with a study using sentences with non-words, i.e. mimicking phonological and morphosyntactic properties of language but without distinguishable semantic information and showing that 11–13 years old children performed much worse than adults, for recognizing emotions from stimuli derived from their native (English) as well stimuli from a foreign languages^46^. In this study, however, the 11–13 years old were not better than 8–10 years old, therefore suggesting no development during late childhood. It may be that the small sample size would have prevented the observation of small differences in performance between 10 and 13 years old; indeed our data show that the maturation curve is less steep than in early childhood. In addition, it is possible that the context of being exposed to pseudo-sentences from different languages adds another level of complexity, which might impact more the pre-and early adolescents, who may automatically search for meaning and thereby be distracted from the task. Indeed it has been shown that when other contextual information is present, young children, but also 13-years old individuals, tend to rely less on prosody and more on semantic information than adults to infer the speaker’s emotion^32^. This might explain differences between studies, but also reflect the fact that vocal emotion discrimination might still be difficult at the beginning of adolescence. The choice of stimulus material and confounds related to different linguistic materials might also explain while some individual studies using sentences as stimuli have reported no change in prosody processing beyond late childhood^4,5,28^.

Our data using non-linguistic vocalizations clearly show that vocal affect categorization progresses throughout childhood and well into adolescence. This developmental timeline is similar to the one reported by many studies of affective facial^4,7–9^ or bodily^61^ expressions recognition. Such changes in social perception abilities are in line with social challenges and refinement that occur during this period of life: as social relationships become more complex, individuals need to be able to detect and categorize subtle social cues more accurately. Improvement in social signals reading also coincides in time with structural and functional changes in brain areas involved in processing emotional signals, such as the amygdala^62^ or the ventral prefrontal cortex^63–66^, as well as posterior cortical areas involved in social cognition, including voice processing^38,42,43^. Additional studies are needed to relate regional changes in brain activity and structure to behavioral changes in emotion processing. Nonetheless the protracted development of social abilities, at the cerebral and behavioural level, is in line with evolutionary theories which propose that the adaptive value of adolescence, as a distinct developmental stage, is to enable the maturation of refined social perception and social skills abilities^67,68^.

The observed female advantage for emotional categorization echoes numerous studies in adults and children focusing on different channels of emotion communication, including faces^7,52,53,69–71^, body movements^72,73^, voice and prosody^49,74–76^. Although robust in meta-analyses^20,21^, this effect is small and not always apparent in individual studies. Its expression and amplitude may depend upon factors intrinsic to the stimuli or contexts^77^. In particular, Sauter *et al*.^29^ and Chronaki *et al*.^27^, who used also non-linguistic affective vocalisations did not report any  statistically significant sex difference in affective voice processing in children, in contrast to our ‘ and Allgood *et al*.’s results. The larger interindividual variability in children, compared to adolescents and adults, may also have masked small differences. In addition, Chronaki *et al*.^27^ used only three emotions, happiness, sadness and anger; in our dataset, the sex effect was larger for fear recognition in the youngest participants, which contributed to the overall statistical significant effect. Interestingly the highest gender differences for fear recognition, as compared to other emotions, mirrors what has been reported in developmental studies of facial affect recognition^10^. Crucially, we observed no interaction between sex and age effects: that is the difference between boys and girls did not change with age. This developmental stability might reflect some evolved differences between males and females present, at least partly, very early in ontogeny. This is in line with earlier meta-analyses of facial or multimodal affective expression recognition^53^ and thus compatible with integrative models suggesting that scaffolding and/or socialisation factors would contribute to maintaining gender differences in non-verbal signal processing that appear during infancy^20^. Moreover the observation of the same developmental trajectory in boys and girls indicates that the improvement in recognizing emotions from vocalizations is independent of pubertal and hormonal changes, which occurs earlier for girls.

Not all basics emotions were identified with equal accuracy. In fact, age-related changes, although significant for all emotions, varied with emotion. Across age groups happiness was the easiest emotion to recognize. This mimics what has been described in adults^49^ and children^27,47^. This is also consistent with the easiest recognition of happiness from facial expressions by adults and children alike^6,10,78^. This high score for happiness might be explained, in part, by the fact that it was the only positive emotion and thus could potentially be categorized using only valence information. The high accuracy of sadness recognition contrasts with previous reports of vocal affect recognition showing that young children had difficulty recognizing sadness^27,47^ and reporting a slow development for the decoding of this emotion. This discrepancy could be related to the nature of the stimuli: in our study, sadness stimuli consisted of relatively salient cries lasting about 2 seconds, and which were also recognized with relatively high accuracy in adults^49^. In contrast, stimuli in Chronaki’s study^27^ were shorter (700 ms) which may have made them more ambiguous and difficult to decipher. In accordance with this hypothesis developmental studies of affective prosody decoding from sentences report high accuracy for sadness in children, pre-adolescents^29,46^ and adolescents^60^.

The two emotions that showed the most significant improvement with age were anger and fear, two negative-valence threat-related emotions. Also, anger proved to be the most difficult emotion to recognize with the oldest adolescents still having lower scores than adults, often miscategorizing angry expressions as sad. This is consistent with some developmental studies of emotion recognition from vocal^47^ or facial expressions^10,78^. However, this finding contrasts with other studies in adults or younger children showing a superior recognition for angry voices as compared to other emotions such as sadness or fear^5,27,29,49^. From a behavioural point of view, in theory, one could argue that a reduced sensitivity to threat signals could promote social exploration and expansion of interpersonal relationships and thus be beneficial in late childhood and adolescence. The poor performance of children for fear recognition and the steeper development for this emotion is striking. It is in line with a couple of developmental studies on processing affective prosody from sentences in children and adolescents^5,46,60^.In addition, meta-analyses indicate that the association between psychopathy and poor fear processing from prosody is stronger in children and adolescents compared to adults^18^. Yet they are based on very few studies and lack comparison data. Our results could serve as normative data to assess vocal emotion recognition, from non-linguistic material, in pathological populations and eventually identify vulnerability periods and/or prognostic markers, for example for psychopathy^18^ or schizophrenia^79^. Likewise, the present study could serve as a starting point to investigate how perceptual (e.g. hearing threshold, or sensitivity to individual acoustic cues), personal or behavioural (e.g. IQ, social anxiety or attention) characteristics could affect vocal emotion recognition across development.

Lastly, we observed the same range of performance as well as the same developmental trajectory in the two groups of participants, namely those tested with sounds presented on individual headphones and response given on keyboard *vs*. those tested with sounds presented on loudspeakers and responses given by circling labels on a pre-formatted paper sheet. Firstly, this signifies that children and adolescents were not influenced by the mode of sound presentation and response while expressing their judgments. Secondly, it shows that data from developmental studies conducted with different procedures, computerized or not, can be directly compared. The same conclusion has been reached in a large scale (n > 1500) study of facial expressions recognition, where strikingly similar developmental trajectories were observed, for all emotions investigated, in sub-samples tested in school on a paper version, in the lab on a computerized version or online^7^. The same conclusion was also apparent in a meta-analysis of developmental studies of facial affect recognition^21^. Thus, the choice of computerized or other conventional laboratory methods should not matter in the design of new developmental studies, thus facilitating the collection of larger datasets.

In summary, our results demonstrate, for the first time, that the ability to identify basic emotions from non-linguistic elementary affect bursts continues to mature from childhood until mid- adolescence. This is akin to what has been described for the recognition of emotion through other channels, like faces and bodies. Insight into this protracted development has implications to characterize adolescents’ interpersonal relationships and to shed light on the development of more complex social competences, such as learning from others or social feedback processing^80^. Charting the normal developmental path of vocal emotion perception has also critical implications for enhancing our understanding neurodevelopmental disorders, like schizophrenia, psychopathy or autism, for which abnormal voice processing has been proposed as an underlying factor of social anomalies^18,81^.

## Methods

### Participants and procedure

Participants were recruited from several after-school clubs, a private school and a college in the greater Glasgow area (Scotland). Permission was obtained from the relevant authorities. Parents as well as children were informed of the purpose of the study and signed a consent form. The study was approved by the University of Glasgow College of Science and Engineering Ethics committee and all experimenters had appropriate disclosure from the Scottish government to work with children. All research was performed in accordance with relevant guidelines from the Declaration of Helsinki, recommendations from the Economic and Social Research council of the United Kingdom and local regulations in particular with regards to data anonymization and storage.

We collected complete datasets from 225 participants (104 males) aged 5–17 (mean 11.87; sd 3.34). Based on existing literature in vocal and facial emotion perception recognition this sample is adequate to detect linear age-related changes as well as gender effects^48,82^ with small to medium effect size. One hundred and thirty-three were tested in small groups (three to 10 participants), with the sounds being presented through loudspeakers and participants reporting their responses on a preformatted sheet of paper with one line per trial. They had to circle or tick, to their choice, the box corresponding to their response. The other 92 participants were tested on individual laptops, with the sounds presented through headphones and responses made through keyboard presses. In both cases the responses were made by selecting one box out of four boxes labeled with the emotions happy, fearful, angry or sad displayed as emoticons on a horizontal line. All participants were familiar with the matching between the emoticons (i.e. facial expression cartoons) and individual emotions. Four different spatial orders were used and counterbalanced across participants to avoid response biases due to the location of the response boxes. All participants completed five practice trials before the experiment. For the youngest participants (<10 years old) the experimenters took extra care in ensuring that the task was well understood and asked them to report a situation in which they would experience each of the emotions to confirm that they knew the meaning of each label. The experimenter stayed close to the children in all cases, monitoring that they had no difficulty selecting the labels (with pencil or keypress) and that they moved smoothly from one trial to the next.

As a control group, we tested 30 adults (age 20–47, 16 males). They were recruited through our local subjects’ pool and tested on individual laptops.

### Stimuli and design

Stimuli were taken from the Montreal Affective Voices databases (MAV^49^). They consist of nonverbal affective bursts uttered by five male and five female speakers selected out of 22 actors to yield maximum recognition agreement amongst young adult listeners. We thus had ten exemplars of each emotion (anger, fear, happiness and sadness) and each participant was presented randomly with five stimuli for each emotion. In a four-alternative forced choice task, they were asked to select the emotion they thought the speaker was expressing.

### Analysis

We assessed the effect of age on the proportion of correct labeling choice with mixed-model logistic regression^83^ chapter^84^. We used the lme4 toolbox (version 1.0–5) in R^85^. We entered each response as a binary variable (i.e. correct or incorrect) into the regression model with random effect “subject” and fixed effects “age” (centered around the sample mean), “sex”, and “testing procedure” (computer or paper) as well as the interaction terms. We tested both linear and non-linear effects of age by adding polynomial terms to the regression model. Significance of main effects or interaction is reported in terms of the Wald statistic. Fixed-effects were also further evaluated using chi-square tests on the log-likelihood values to compare models with and without the effect of interest. From the model yielding the best fit, we computed the predicted percent correct responses at each age by inverting the logit link using the “fitted” function in R.

For additional description of the data we grouped the participants in four different age-groups, which we also compared to the adults group: children: 5–8 years old, n = 41 (25 females); early adolescents: 9–11 years old, n = 67 (28 females); mid-adolescents: 12–14 years old n = 54 (30 females); late-adolescents: 15–17 years old, n = 63 (38 females). There was no significant difference in gender balance between the groups (Chi-square = 5.8799, df = 3, p-value = 0.1176). For all comparisons between groups we used the Holm-Bonferonni method to account for multiple comparisons. We also computed confusion matrices in these age groups, reflecting the patterns of misinterpretation amongst the different emotions. That is, for each emotion portrayed we computed the number of times it was misclassified for each of the other emotions.

The datasets generated during the current study are available from the corresponding author upon request.
